# Enhancing the early detection of Alzheimer’s disease using an integrated CNN-LSTM framework: A robust approach for fMRI-based multi-stage classification

**DOI:** 10.1371/journal.pone.0317968

**Published:** 2025-08-26

**Authors:** Saima Farhan, Yasin Ul Haq, Momina Abdul Khaliq, Seemal Afza, Fahad Ahmad, Tariq Mahmood, Amjad Rehman

**Affiliations:** 1 Department of Computer Science, Lahore College for Women University, Lahore, Pakistan; 2 Department of Computer Science and Engineering, University of Engineering and Technology Lahore Narowal Campus, Narowal, Pakistan; 3 School of Computing, Faculty of Technology, University of Portsmouth, Southsea, Portsmouth, United Kingdom; 4 Artificial Intelligence and Data Analytics (AIDA) Lab, CCIS, Prince Sultan University, 11586, Riyadh, Kingdom of Saudi Arabia; 5 Faculty of Information Sciences, University of Education, Vehari Campus, Vehari, Pakistan; National University of Sciences and Technology, PAKISTAN

## Abstract

Alzheimer’s Disease poses a significant challenge as a progressive and irreversible neurological condition striking the elderly population. Its incurable nature correlates with a significant rise in death rates. However, early detection can slow its progression and facilitate prompt intervention, thereby mitigating mortality risks. Functional Magnetic Resonance Imaging (fMRI) provides valuable insights into the functional changes within distinct brain regions associated with the disease. The recent research efforts have extracted functional connectivity measures for the classification. These handcrafted functional connectivity features are usually not robust and are computationally intensive. To address the issue, this study introduces an integrated deep-learning framework based on CNN and LSTM networks. This framework autonomously learns both intra-volume and inter-volume features critical for classification tasks. CNNs facilitate feature extraction, while LSTM networks govern the selection of significant features for classification. The key aim of this study is to classify Alzheimer’s disease and its prodromal stage, Mild Cognitive Impairment (MCI). MCI is further categorized as early MCI (EMCI) and late MCI (LMCI). We have evaluated the framework in three dimensions, binary classification, multi-class classification with 3-classes, and multi-class classification with 4-classes. For each dimension, multiple classifications were performed. The results depict the proposed CNN-LSTM framework to attain 99% accuracy and 100% average area under the curve for the majority of the classification.

## Introduction

Alzheimer’s Disease (AD) is characterized by a progressive and irreversible degeneration of tissues and nerve cells across various brain regions, leading to neurodegeration. The irreversible damage to the brain tissues causes a gradual deterioration of a larger volume of the brain, ultimately impairing the patient’s cognitive abilities like thinking, memorizing, and decision-making. A mild deterioration of brain cells accountable for thinking and memorizing steadily leads to dementia, which refers to the complete loss of cognitive abilities [1]. At the early stage of the disease, an individual experiences difficulty in thinking, rational reasoning, and registering new memories in the brain, and over time the patients become completely incapable of carrying out their day-to-day tasks. AD appears with age, consequently, most individuals aged 50-90 are at a higher risk [2]. Additionally, age isn’t solely responsible for AD, distinctive variables like environment and family history also play a vital role in its progression.

AD not only affects an individual’s quality of life but can also lead to fatalities in severe cases. Official death certificates recorded 119,399 deaths from AD in 2021 [3]. The mortality rate among AD patients has increased significantly by 140% from 2000 to 2021, thereby ranking it as the fifth leading cause of death worldwide [4]. Currently, approximately 90 million individuals are affected by AD, with projections indicating an increase to 13.8 million by 2050 [5].

As a result of the elevated death rate, early diagnosis of the disease is a dire need of time, which would be highly helpful to slow down its progression towards AD and might preserve the cognitive functions of the brain as well. Mild Cognitive Impairment (MCI), recognized as a transitional phase between AD and Cognitively Normal (CN) has gained significant attention in recent times [6]. MCI is the prodromal stage of AD marked by cognitive decline that does not adversely affect an individual’s thinking and memory abilities but poses a heightened chance of progressing to AD [7]. MCI is further divided into two stages i.e. Early Mild Cognitive Impairment (EMCI) and Late Mild Cognitive Impairment (LMCI) [8].

A thorough clinical assessment, encompassing the patient’s medical history and psychometric evaluation tests such as the Clinical Dementia Rate (CDR), Mini-Mental State Examination (MMSE), and Functional Activities Questionnaire (FAQ) is necessary for the diagnosis of AD. These assessment methods are time-consuming and unable to diagnose the disease at early stages. However several brain imaging techniques help in the early diagnosis of the diseases. These imaging techniques are further split into two groups, i.e. structural and functional imaging. Structural imaging is used for capturing anatomical changes, like in AD, it helps detect the deteriorating volume of the brain. Widely used structural modalities include structural Magnetic Resonance Imaging (sMRI) and Computed Tomography (CT) [9]. However, functional evaluation of the brain is necessary for early illness prediction because physical alternations in the brain only happen in the later stages of AD. Functional evaluation is done using functional imaging techniques including Electroencephalography (EEG), functional Magnetic Resonance Imaging (fMRI), Magnetoencephalography (MEG), Positron Emission Tomography (PET), and Single-Photon Emission Computed Tomography (SPECT) [10,11]. Due to the advantage of high spatial and temporal resolution, fMRI is a widely-used modality [12].

Machine Learning (ML) has the potential to learn details about data by performing feature engineering and making predictions on new data of the same class without the need for explicit instructions [13,14]. Various ML techniques have been applied to different neuroimaging modalities for AD detection. The most prominent of these choices is Support Vector Machine (SVM). An SVM-based computer-aided framework is employed on SPECT data which extracts the statistical features through Normalized Mean Squared Error (NMSE) for early AD detection. In this framework, 20 features are selected using a t-test through weighting feature correlation and are further classified using linear kernel SVM [15]. Another Computer-Aided Diagnosis (CAD) system has been employed for the prediction of conversion from MCI to AD. Feature vector formation is a two-step process; segmentation of Volume of Interest (VOI) followed by extraction of the voxel value of segmented VOI. A subset of optimal features is selected based on feature ranking using a t-test score and genetic algorithm. Subsequently, SVM is used for disease prediction [16]. An ensemble classification framework is employed for the identification of MCI and AD, where various subsets of features are presented to each base classifier which are SVM and Random Forest (RF) [17]. A combination of SVM and graph theory is developed for the classification of fMRI data into AD, MCI, and CN [18].

Various studies have combined multiple modalities for AD-MCI classification using RF by utilizing a unified graph. To create this graph, commonalities between MRI volumes, category genetic data, voxel-based PET signal intensities, and Chronic Fatigue Syndrome (CFS) biomarker measurements are extracted [19]. Some approaches have utilized MRI and PET data either for AD diagnosis [20] or for finding disease progression of Mild Cognitive Impairment Converters (MCI-C) and non-converters (MCI-NC) to AD patients [21]. SVM has been employed to anticipate the conversion of MCI to AD patients after integrating sMRI and fMRI data [22]. Despite its remarkable potential in automation, ML has been severely criticized for its handcrafted feature extraction and selection. This process requires a significant amount of domain understanding and considerable manual effort.

Deep Learning, a subfield of machine learning, can automatically learn optimal features, thereby reducing the need for manual efforts [23]. A variety of studies have utilized deep learning methodologies for classifying neuroimaging data. One of the widely adopted deep learning methods is the Convolutional Neural Network(CNN). The variants of CNN architecture have also been employed for the classification of multiple classes of AD including moderate AD, mild AD, and very mild AD [24]. For the diagnosis of AD, researchers mostly analyze MRI data with the help of 3D CNNs [24–27]. Additionally, pre-trained and transfer learning-based models like VGG, GoogleNet, and inception V3 have also been implemented for the early detection and conversion prediction of MCI to AD patients. Furthermore, for classifying AD and different stages of MCI, a set of discriminative features extracted through the use of unsupervised learning have been incorporated into an ensemble classifier which is composed of a Deep Belief Network (DBN) used as an underlying classifier but the final prediction is determined through voting [28].

This paper presents an integrated model for early detection of AD by utilizing fMRI modality. The data is acquired from a publicly available repository. Preprocessing is performed on the acquired dataset. For classification, the initial stack of intra-volume features is extracted using CNN without a softmax layer and the inter-volume features are extracted subsequently by using Long Short Term Memory (LSTM). Finally, the softmax layer, followed by dropout and dense layers, is used to classify each participant. The goal of this research is to create an effective method that implicitly predicts the disease at an early stage, which is advantageous for lowering the mortality rate of AD patients and slowing down its progression by developing advanced computer-aided techniques.

Our proposed CNN-LSTM model distinguishes itself from traditional neuroimaging approaches in several ways. By integrating both CNN and LSTM components, this framework captures intra-volume features within each fMRI scan (through CNN layers) and sequential inter-volume features across multiple scans (through LSTM layers). This dual approach enables the model to process both spatial and temporal data effectively, which is crucial for accurately distinguishing between stages of Alzheimer’s Disease and its prodromal stages. Additionally, our study addresses the multi-stage classification of AD, including classifications of MCI into EMCI and LMCI, as well as AD itself. Achieving high classification performance across binary, three-class, and four-class tasks, our model contributes to a more nuanced understanding and identification of early-stage cognitive impairment, an area less explored in the literature. This framework demonstrates the potential for a practical, early diagnostic tool that offers a robust and detailed approach to AD staging.

The paper is structured as follows: it begins with the materials and methods section that are employed during this study, followed by the presentation of results and validation. Next, the discussion includes the comparisons with related research work, and finally the paper concludes with the summary of key findings.

## Materials and methods

### Materials

The data used in this study is sourced from the Alzheimer’s Disease Neuroimaging Initiative (ADNI) database, which is accessible to researchers upon request. To access the ADNI data, researchers can visit the ADNI website at http://adni.loni.usc.edu/ and follow the data access request procedures outlined there. Access to the ADNI database is granted for research purposes and requires approval of an application that details the intended research use. It comprises multiple neuroimaging datasets for different subjects belonging to the groups AD, LMCI, MCI, EMCI and CN. Requests for data access should be directed to http://adni.loni.usc.edu/data-samples/access-data/.

The Motion Corrected (MoCo) series-based fMRI subjects were selected for this study consisting of 413 participants. All participants further fall into the following groups: 140 CN, 66 AD, 34 MCI, 93 EMCI, and 80 LMCI subjects. The comprehensive clinical and demographic information of the participants is provided in Table 1.

**Table 1:** The clinical and demographic information of participants. SD: Standard deviation, CDR: Clinical Dementia Rating, MMSE: Mini-Mental State Examination.

Group	Participants	Age	Gender (M/F)	CDR Score (Mean ± SD)	MMSE Score (Mean ± SD)
AD	66	56-88	37/29	0.92 ± 0.31	21.24 ± 3.44
CN	140	55-91	56/84	0 ± 0	29.52 ± 0.5
MCI	34	55-90	19/15	0.50 ± 0.17	27.67 ± 1.8
EMCI	93	56-85	49/44	0.45 ± 0.22	28.10 ± 1.57
LMCI	80	55-85	39/41	0.54 ± 0.14	27.11 ± 2.44

#### Ethical considerations in the use of human data.

This study utilizes data from the ADNI, a publicly accessible dataset collected under strict ethical guidelines. ADNI obtained informed consent from all participants, allowing their data to be used in research aimed at advancing understanding and treatment of AD. To protect participant privacy, the ADNI dataset is fully anonymized and adheres to data privacy standards. As secondary users of this dataset, we ensured that our use of the data aligns with ADNI’s ethical protocols, including respecting participant consent and data handling guidelines. Researchers accessing this dataset are required to comply with ADNI’s terms of use, ensuring that participant data is handled with the utmost ethical responsibility. Our study thus upholds ethical standards in both data usage and participant privacy.

### Methods

**Fig 1:**
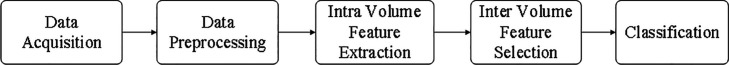
The overall architecture of the proposed study.

The proposed research comprises five major modules. The first module is data acquisition in which fMRI-based volumetric images are obtained. The second module is data preprocessing which includes the conversion of Digital Imaging and Communications in Medicine (DICOM) format into Joint Photographic Extension Group (JPEG) format after discarding initial volumes, resizing the images, and formation of VOI against each subject. The third module is the intra-volume feature extraction that employs multiple layers of CNN, excluding the activation function in the final layer. The fourth, and crucial module is inter-volume feature extraction which specifically deals with time-series image data using LSTM layer. The last module is the classification of selected features using the softmax activation function. The entire process of the proposed research is illustrated in Fig 1.

#### Data acquisition.

The fMRI scans selected for the proposed research are acquired using SIEMENS scanners with Verio model and the field strength is 3.0 tesla. For each participant, there are 105 volumes, 24 slices with slice thickness of 4.0mm, the Echo Time ranging from 12.0ms to 13.0ms, the Repetition Time of 3400ms, the flip angle of 90 degrees, the matrix size of 320×320 pixels, the pixel spacing of 4.00mm in X and Y dimension and the pulse sequence.

#### Data preprocessing.

Recent studies have investigated deep learning applications in medical image classification, focusing on the impact of different image formats. While DICOM is the standard for medical imaging, conversion to formats like JPEG is common for compatibility with deep learning frameworks. For example, Chiang et al. (2021) demonstrated that CNNs can accurately classify medical images by modality and anatomical location using JPEG format, achieving excellent accuracy [29]. Kim & Kim (2022) found that classification performance was unaffected when converting DICOM images to TIFF, PNG, or JPEG formats [30]. However, Maruyama et al. (2018) noted that traditional machine learning methods like SVM and Artificial Neural Network (ANN) showed decreased accuracy with JPEG compared to DICOM, while CNN maintained high accuracy for both formats [31].

The selection of 224 × 224 pixels for image scaling in deep learning applications balances computational efficiency and spatial resolution. This dimension is commonly used in CNNs for medical imaging, including neuroimaging studies [32].

**Fig 2:**
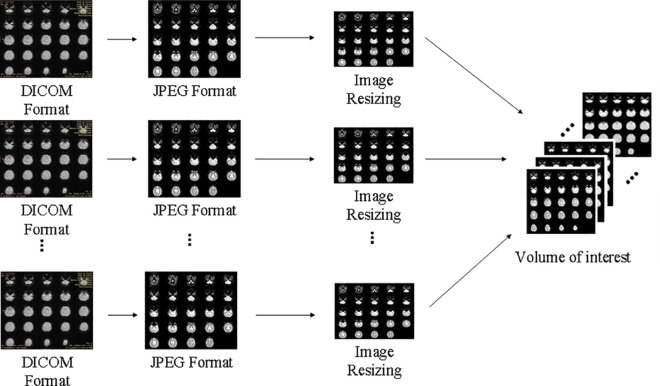
Data preprocessing steps.

Our data preprocessing pipeline consists of the following four steps for each subject: (i) The DICOM images are converted to JPEG format. Although DICOM contains metadata or additional information, our primary concern is with pixel data only for feature extraction. Therefore, DICOM data is converted to JPEG as it provides a good balance between compression and image quality and is compatible with deep learning frameworks and image processing libraries. (ii) For signal equilibrium, first 15 volumes of each subject are discarded and the rest of the 90 volumes are utilized for the quantification of the proposed study. (iii) Subsequently, the remaining volumes are resized to 224 × 224 to acquire consistent dimensions between the volumes of each subject. The 224 × 224 dimension is a commonly used standard in deep learning models. (iv) Finally, for the formation of VOI, all the volumes of the subjects are stacked up one by one sequentially, as shown in Fig 2. The characteristics of the selected dataset for the evaluation of the proposed research are shown in Table 2.

**Table 2:** Detail description of selected dataset.

Participants	Groups	Volume per Participants	Slices per Vol.	Format	Dimension
413	5	90	24	JPEG	224×224

#### Proposed model.

The primary objective of the proposed research is to successfully classify fMRI scans, but the major issue is that the fMRI scan is a time series data that contains multiple volumes against a single subject. Due to this reason, the proposed approach employs the following steps: (i) Intra-volume feature extraction, (ii) Inter-volume feature selection, and (iii) Classification. Intra-volume features are the type of features that are learned through a single volume of a participant, but inter-volume features are learned from multiple volumes of a single participant.

**Intra-volume feature extraction.** CNN is a multilayer neural network, which implicitly learns the features from images at the pixel level, however, the conventional neural networks had to rely on handcrafted features. For object detection, an image classification CNN is a widely utilized deep neural network because it reduces the number of hyperparameters and is robust to noise. CNN is primarily proposed to extract the intra-volume features. There are multiple variants of CNN architecture proposed by many researchers recently. Every CNN variant is composed of multiple layers including convolution, activation, normalization, max pooling, flattening, and softmax layer with different numbers of parameters [33]. In the proposed research, an 18-layered CNN architecture is employed, consisting of convolution, ReLu, max-pooling, dropout, and flattened layers.

In the CNN, the initial layer is the convolution layer, responsible for extracting the feature map by convolving the learned filter over the input image. Feature maps contain the discrimination information of the image that is activated when the convolutional filter and patch of the input image meet the criteria. An activation function, such as tanh, sigmoid, or Rectified Linear Unit (ReLu), follows each convolutional layer. Subsequently, the next layer performs max pooling which down-samples the feature maps by only keeping the most influencing and distinguishing features. To address the issue of overfitting, the output of the max pooling layer serves as the input to the dropout layer. The next one is the flattened layer which reduces the feature map dimensions into a single-column feature vector (FV). Finally, a softmax layer is added for the classification of FV.

The proposed CNN architecture comprises an input layer, 5 convolution layers, 5 ReLu layers, 5 max-pooling layers, a dropout layer, and a flattened layer. The other parameters of the network are listed in Table 3. The kernel size for the convolutional layer is fixed to 3×3 but a different number of filters are set from 32, 64, 128, 256, and 512 respectively. Following each convolution layer, there is an activation layer utilizing ReLu as the activation function along with a max pooling layer with a kernel size of 2×2. Subsequently, a dropout layer with a rate of 0.25 is pipelined followed by a flattened layer at the end, as depicted in Table 3.

**Table 3:** The CNN architecture and its parameters.

Layer ID	Layer Name	No. of Kernels	Kernel Size	Output Shape
1	Input Layer			224x224x1
2	Convolution	32	3x3	222x222x32
3	Relu			222x222x32
4	Max Pooling		2x2	111x111x32
5	Convolution	64	3x3	109x109x64
6	Relu			109x109x64
7	Max Pooling		2x2	54x54x64
8	Convolution	128	3x3	52x52x128
9	Relu			52x52x128
10	Max Pooling		2x2	26x26x128
11	Convolution	256	3x3	24x24x256
12	Relu			24x24x256
13	Max Pooling		2x2	12x12x256
14	Convolution	512	3x3	10x10x512
15	Relu			10x10x512
16	Max Pooling		2x2	5x5x512
17	Dropout(0.25)			5x5x512
18	Flatten			12800

**Fig 3:**
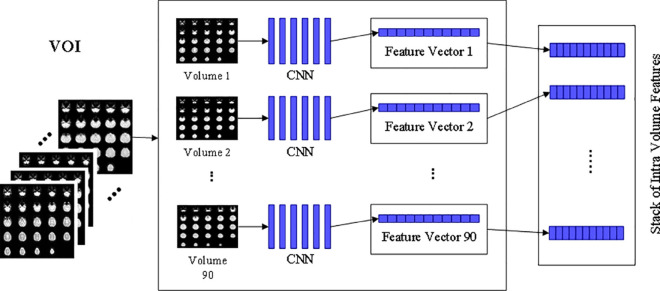
Intra volume feature extraction.

For intra-volume feature extraction, one volume of a subject is extracted from the VOI and is passed to CNN for generating a feature map before extracting the next volume and its FV, the same process continues until the FV are obtained for all 90 volumes. The complete procedure of inter-volume feature extraction is presented in Fig 3.

**Inter-volume feature extraction.** Each fMRI scan consists of multiple volumes for a single subject. The classification of these scans requires feature extraction, specifically inter-volume features. Although CNNs lack the capability to classify sequential data of participants, Recurrent Neural Network (RNN) proves proficient in this task [34]. RNNs use a loop mechanism to propagate information to the subsequent time steps, enabling the network to retain and process sequential data. The current output is influenced by prior learning iterations, thereby facilitating operations over sequences of vectors.

RNNs possess short-term memory, which retains pertinent input information and facilitates output generation, subsequently looping back the output for the next network step. Weight assignments to both current input and output from the preceding step maintain this information, with updates dictated by gradients and error [35]. Gradients govern weight updates but may encounter challenges such as vanishing or exploding gradients, both of which impede RNN performance [36,37]. While exploding gradients can be mitigated by gradient truncation, resolving vanishing gradients proves more complex. However, LSTM offers a solution.

LSTM extends RNN functionality by preserving information over more extended periods through memory retention [38]. By regulating the opening and closing of gates, LSTM can retain or discard information effectively. comprising input, forget, and output gates, LSTM determines the storage, erasure, and passing of information, respectively. Determining the prediction time steps is crucial for LSTM after feature extraction, particularly in processing 90 intra-volumes as a sequence. Post-CNN feature extraction, LSTM processes a stack of 90 intra-volume feature maps for each participant, establishing sequential correlations between consecutive volumes.

**Fig 4:**
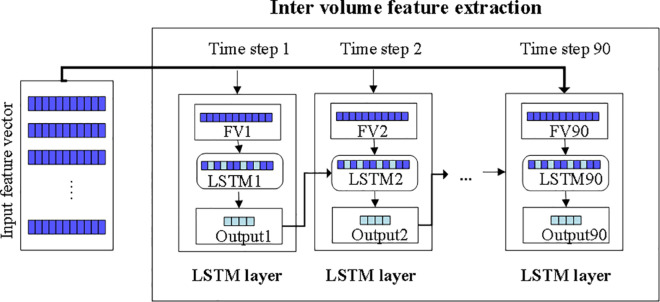
Inter volume feature extraction using LSTM layer, FV: feature vector.

The proposed model, at time step t1, inputs a FV from the stack, selects relevant features, and loops back these learned features. At time step t2, selected features depend on current FV stack elements and those from the preceding time step. This iterative process continues until all 90 intra-volume features are processed, ultimately yielding inter-volume features. The comprehensive procedure of inter-volume feature extraction is presented in Fig 4.

#### Classification.

In this study, hyperparameters were selected based on preliminary experiments and established best practices, rather than through systematic optimization. We empirically chose a dropout rate of 0.25, a learning rate of 0.001, and a batch size of 32. Each hyperparameter choice was made to balance performance and computational efficiency, with the following observations:

**Dropout Rate:** A dropout rate of 0.25 was found to effectively balance generalization and model capacity. Lower dropout rates (e.g., 0.1) led to minor overfitting, while higher rates (e.g., 0.4) reduced accuracy due to excessive regularization and potential information loss.

**Learning Rate:** The selected learning rate of 0.001 allowed stable convergence. Higher rates (e.g., 0.005) led to fluctuations in the loss function, while lower rates (e.g., 0.0001) extended training time without substantial gains in performance.

**Batch Size:** A batch size of 32 offered a practical balance between training speed and stability. Smaller batch sizes (e.g., 16) improved accuracy marginally but increased computational requirements, while larger sizes (e.g., 64) led to slight decreases in accuracy.

In future work, we intend to explore systematic hyperparameter tuning using techniques such as grid search, random search, or automated tuning libraries (e.g., Optuna) to enhance model performance further.

Following the LSTM layer, a dropout layer with a dropout rate of 0.25 is incorporated for classification purposes, serving to alleviate concerns related to over-fitting. Subsequently, a dense layer is employed, establishing connections between individual output neurons and the softmax layer, which serves as the conclusive layer in the classification model.

## Results

This research proposes a CNN-LSTM-based framework to analyze fMRI data for AD and its prodromal stages MCI, EMCI, and LMCI. The framework involves three phases; intra-volume feature extraction, inter-volume feature extraction, and classification of inter-volume features. Evaluation of the framework employs data acquired from the ADNI dataset, comprising 413 participants, including 66 AD, 34 MCI, 80 EMCI, 93 LMCI, and 140 CN.

To address the potential biases, the study employs stratified K-fold cross-validation with ten folds [39]. In each fold, 80% of the subjects’ data is allocated to the training set and 20% to the validation set. This 80:20 division was carefully designed to prevent any overlap between training and validation subjects. Each fold participates as a validation set once, whereas the remaining k-1 folds serve as the training set. In this way, each fold gets a chance to participate in training at least once, thereby safeguarding against information leakage and ensuring the integrity of model performance results. Evaluation metrics include accuracy (ACC), sensitivity (SEN), specificity (SPE), average Area Under the Curve (AUC), confusion matrix, and Receiver Operator Characteristic (ROC) Curve [40–42]. The classification process utilizes an Adaptive Moment Estimation (Adam) optimizer with a batch size of 32 [43].

The experimental setup comprises three levels of classification. At the first level, binary classification is performed. The second level involves multi-class classification with three distinct classes, followed by the third level which undertakes multi-class classification with four classes, as outlined comprehensively in Table 4. Given the variation in the number of participants across groups, Class Balancing is performed to mitigate potential biases.

**Table 4:** Classification results based on three levels of experiments, binary classification, multi-class classification with 3-class and 4-class.

Classes	Class Specification	Instances	ACC (%)	SEN (%)	SPE (%)	AUC (%)
2	CN vs EMCI	186	99.03	99	99	100
	EMCI vs LMCI	160	99.67	99	99	100
	LMCI vs AD	132	94.87	94	94	94
	CN vs AD	132	99.91	99	99	100
	CN vs LMCI	160	99.05	99	99	100
	CN vs MCI	68	99.64	99	99	100
	MCI vs AD	68	99.20	99	99	100
	EMCI vs AD	68	99.06	99	99	100
3	CN vs MCI vs AD	102	98.99	98	98	100
	CN vs EMCI vs LMCI	198	94.37	94	94	98
	EMCI vs LMCI vs AD	198	99.01	99	99	100
4	CN vs EMCI vs LMCI vs AD	264	99.26	99	99	99.50

There are two distinct stages of MCI: EMCI and LMCI. It is unnecessary to classify the disease versus the stage of the same disease. At the initial level, each group is compared with all other groups, resulting in ten combinations. However, two combinations, namely MCI vs EMCI and MCI vs LMCI, are logically invalid.

**Fig 5:**
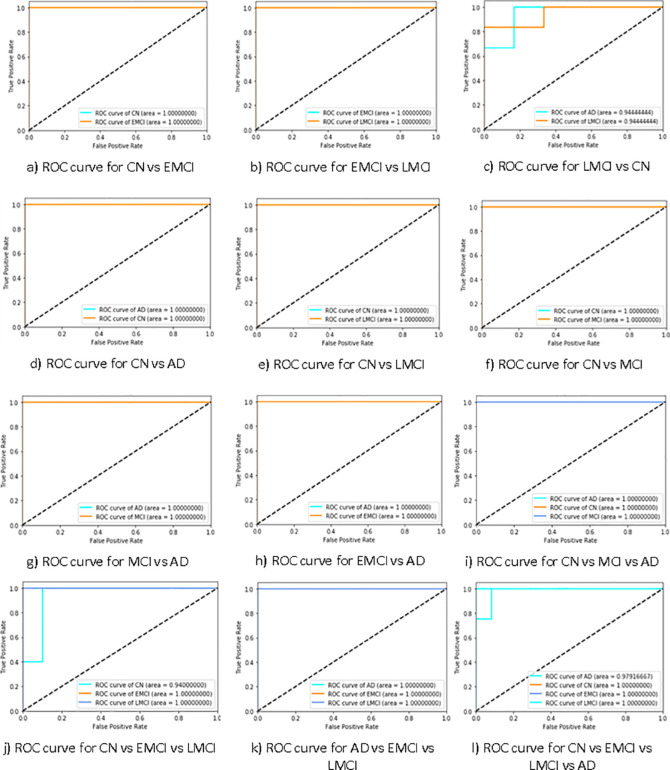
ROC curves of all classification, a) to h) represent the binary classification, i) to k) represent the 3-class classification, and l) represent the 4-class classification.

For binary classification, eight different combinations of groups are considered, including CN vs EMCI, EMCI vs LMCI, LMCI vs AD, CN vs AD, CN vs LMCI, CN vs MCI, MCI vs AD, and EMCI vs AD. The proposed model yields 99% accuracy with an AUC of 100% for all combinations except for the LMCI vs AD, which attains 94% accuracy and AUC. The ROC curves of binary classification across all combinations are depicted in Fig 5.

At level two, multi-class classification is performed, focusing on three classes at a time. Valid combinations in this phase include CN vs AD vs MCI, CN vs EMCI vs LMCI, and AD vs EMCI vs LMCI. A total of 102 participants were successfully classified as AD, MCI, and CN, achieving an accuracy of 98.99% and AUC of 100%.

The three-class classification among CN, EMCI, and LMCI yields an accuracy of 94.37% with an AUC of 98%, while for the combination of AD vs EMCI vs LMCI, the accuracy and AUC are 99.01% and 100% respectively. The ROC curves for three-class multi-class classification are shown in Fig 5.

At the third level, multi-class classification with four classes is performed, focusing on the combination of CN, AD, EMCI, and LMCI. As discussed earlier, EMCI and LMCI are subcategories of MCI, rendering distinctions between MCI stages unnecessary. The four-class CNN-LSTM model successfully achieved a remarkable accuracy of 99.26% with an AUC of 99.50%. The ROC curve for level 3 is shown in Fig 5.

During the model development process, we conducted initial experiments with conventional machine learning techniques and simpler deep learning architectures (CNN-only). However, the results from these baseline models were less significant compared to the optimized CNN-LSTM model. Consequently, we did not retain these results for reporting in the manuscript.

In this study, we focused on the results achieved by the CNN-LSTM model, highlighting its improved accuracy and robustness. Nevertheless, we acknowledge that reporting baseline results would have provided a clearer benchmark for evaluating the improvements introduced by our approach.

In future work, we aim to retain and report detailed results from baseline models, such as SVM and CNN-only architectures, to offer a more comprehensive comparison and further validate the enhancements introduced by the CNN-LSTM approach. These comparisons will strengthen the evaluation of the proposed model’s effectiveness and its applicability for Alzheimer’s Disease classification tasks.

## Discussion

This paper presents a CNN-LSTM-based framework for binary and multi-class classification using fMRI data. To evaluate the proposed framework, fMRI-based dataset is acquired from ADNI data collection, comprising five groups: AD, CN, MCI, EMCI, and LMCI. The classification process includes four modules: (i) data preprocessing, (ii) intra-volume feature extraction, (iii) inter-volume feature extraction, and (iv) classification.

The data preprocessing stage begins with the conversion of DICOM format to JPEG format. The initial 15 volumes of each subject are discarded, and the images are resized to dimensions of 224×224. Finally, VOI formation is performed for each subject. Once the data is preprocessed, our proposed CNN-LSTM framework first utilizes CNN to extract the intra-volume spacial features from the fMRI data, and generates a stack of intra-volume features for each subject. This spatial feature extraction is critical for enhancing diagnostic precision by capturing essential intra-volume details. Subsequently, LSTM extracts the inter-volume temporal features by recurrently analyzing the stack of intra-volume features. This recurrent analysis enables the model to discern subtle, progressive changes in brain activity over time, which is particularly valuable for detecting early indicators of AD. By integrating both intra-volume and inter-volume feature extraction in a single model, this CNN-LSTM framework advances neuroimaging analysis with an automated, dual-layer feature learning mechanism that captures complex spatial-temporal patterns. Finally, the Classification is performed using a softmax layer, along with dense and dropout layers.

Various approaches have been used recently to diagnose AD at an early stage, including binary and multi-class classification using machine learning or deep learning frameworks. Table 5 summarizes the recently developed strategies for binary classification, and Table Table 6 summarizes multi-class classification approaches.

**Table 5:** Comparison of the proposed method with recent approaches for binary classification.

Reference	Subjects	Classes	Method	ACC	SEN	SPE	AUC
Bi et al. [44]	61	AD vs CN	RNN Cluster(Elman-NN)	92.31	-	-	-
Bie et al. [45]	60	AD vs CN	SVM Cluster with K=370	94.44	-	-	-
khazaee et al. [46]	40	AD vs CN	SVM	100	-	-	-
khazaee et al. [22]	168	HC vs AD + MCI	Multi Class SVM	87.29	35	97.96	-
		AD vs HC + MCI			97.96	35	
				97.46	100	70	-
		MCI vs HC + AD			70	100	
				72.03	84.91	61.54	-
					61.54	84.91	
Nguyen et al. [48]	95	AD vs CN	ELM	98.86	100	97.50	-
		MCI vs CN		98.57	100	97.50	-
Sarraf et al. [49]	144	AD vs CN	LeNet and GoogleNet	99.90	-	-	-
Qureshi et al. [27]	133	Very Mild to Mild vs Moderate to Severe	3D-CNN	92.30	89.6	94.6	-
Hojjati et al. [21]	80	MCI-C vs MCI-NC	K SVMs where K=8	93.00	-	-	-
Pei et al. [50]	36	EMCI vs LMCI	SVM	70.00	-	-	70.88
Bi et al. [51]	105	EMCI vs LMCI	Weighted Random SVM Cluster	90.00	90.9	88.89	-
		LMCI vs AD		88.89	85.71	90.9	-
Proposed Approach	132	AD vs CN	CNN-LSTM	99.91	99	99	100
	68	MCI vs CN		99.64	99	99	100
	68	MCI vs AD		99.20	99	99	100
	160	EMCI vs LMCI		99.67	99	99	100
	132	LMCI vs AD		94.87	94	94	94

**Table 6:** Comparison of the proposed method with recent approaches for multi-class classification.

Reference	Subjects	Classes	Method	ACC	SEN	SPE	AUC
Khazaee et al. [22]	168	CN vs MCI vs AD	Multi Class SVM	88.42	70	100	-
					96.92	73.33	
					70	96.47	
Khazaee et al. [47]	168	CN vs MCI vs AD	Naïve Bayes	93.30	88.4	100	-
					100	85.5	
					81.8	100	
Tufail et al. [52]	293	CN vs MCI vs AD	3D-CNN	59.73	-	-	-
Jie et al. [53]	174	CN vs EMCI vs LMCI vs AD	wck-CNN	57.00	-	-	-
Kazemi et al. [54]	197	CN vs SMC vs EMCI vs LMCI vs AD	AlexNet	97.63	-	-	94.86
							93.34
							94.91
							95.00
							94.22
Dar et al. [55]	300	CN vs MCI vs EMCI vs LMCI vs AD	MobileNet	96.22	-	-	-
Proposed Approach	102	CN vs MCI vs AD	CNN-LSTM	98.99	98	98	100
	264	CN vs EMCI vs LMCI vs AD		99.26	99	99	99.50

A study performed cluster-based classification by utilizing a Random Neural network cluster as a feature selector and classifier. At first, the traditional steps of preprocessing were performed on fMRI dataset, then 4005 functional connectivity (FC) of the brain were extracted as features. That research also compares five Neural Networks (NNs) including Back Propagation (BP) NN, Elman NN, Probabilistic NN, Learning Vector Quantization (LVQ) NN, and Competitive NN. However, Elman NN was selected as a base classifier and achieved a higher accuracy of 92.31% by randomly selecting 180 features [44]. Another study employed random cluster analysis with SVM for AD identification that utilized Kernel SVMs (k-SVMs) as base classifiers and the k was set to 370. The Pearson Correlation Coefficient (PCC) was applied to preprocessed fMRI data to extract FC as features. Furthermore, the random SVM cluster yielded an accuracy of 94.44% using 170 optimal selected features [45].

For AD diagnosis, SVM was employed over the graph measures of fMRI data. This study consists of three phases; feature extraction, feature selection, and classification. Firstly, the dataset was preprocessed followed by graph construction for each subject, and calculation of graph measures, distinct features were extracted. By employing the Forward Sequential Feature Selection (FSFS) algorithm, significant features were selected and passed to SVM for classification, which accurately performed binary classification of AD vs CN with 100% accuracy [46].

The same approach was employed for the MCI, AD, and Healthy Control (HC) classification. It involves several steps: (i) preprocessing, (ii) 264 Region of Interest (ROI) extraction using functional area atlas, (iii) formation of functional connectivity matrix, (iv) graph construction, (v) calculation of graph measures, (vi) feature selection using FSFS algorithm, and (vii) classification by SVM. The study not only performed multi-classification with an accuracy of 88.4% but also performed a binary classification of a class from two other classes. For binary classification, it achieved the accuracies of 87.3, 97.5, and 72.0 for HC vs AD and MCI, AD vs HC and MCI, and MCI vs HC and AD respectively [22].

Another study explored a directed graph measuring base features extracted from fMRI for the AD, MCI, and HC classification. Here, Naïve Bayes yielded an accuracy of 93.3%, using features extracted during the Filter and wrapper feature selection phase. FSFS and Sequential Feature Selection (SFS) algorithms were utilized in the filter and wrapper feature selection phase, respectively [47]. For the automatic distinguishing of AD, and MCI from CN, an Extreme Learning Machine (ELM) model was integrated with hybrid Multi Variant Pattern Analysis (MVPA). It consisted of SVM-Recursive Feature Elimination (SVM-RFE), Least Absolute Shrinkage And Selection Operator (LASSO), and univariate t-test in combination. The first step of the study was to extract ten biomarker measures of the processed fMRI, which can be classified into spontaneous regional measures and functional connectivity measures. The ELM was evaluated on two different datasets; ADNI and in-house. For the ADNI dataset, the accuracies achieved for AD vs CN and MCI vs CN were 98.86% and 98.57%, respectively. The in-house dataset yielded 98.70% accuracy for AD vs CN and 94.16% accuracy for MCI vs CN [48].

Instead of employing connectivity networks, CNN can be utilized for the binary classification of AD vs CN. Two distinct pre-trained CNN-based frameworks were proposed for handling fMRI and sMRI data for the subject level and binary classification. Three major steps were carried out for the identification: (i) preprocessing, (ii) data conversion, and (iii) classification. First, both fMRI and sMRI data were preprocessed, and then 2-D images were extracted from the preprocessed data. Subsequently, classification was performed utilizing CNN architectures LeNet and GoogleNet. For subject-level classification, a decision-making algorithm was employed that makes predictions based on majority voting, which means for each subject, the class with more slices was chosen. For binary classification, it yielded the accuracies 99.9% and 98.84% for fMRI and sMRI data respectively, however, concerning the classification of subject level, 97.77% for fMRI and 100% for sMRI accuracies were obtained [49].

A 3D-CNN architecture was employed on fMRI data for the automatic evaluation of the severity of dementia based on CDR score. For that purpose, two groups of AD participants were formed; very mild to mild and moderate to severe. The first group includes all subjects with CDR 0.5 - 1 and the second group consists of all subjects with CDR scores 2 - 3. The Independent Component Analysis (ICA) was employed to extract the FC-based features. The 3D-CNN performs an automatic assessment of dementia severity with an accuracy of 92.30% [27]. Another study finds the progression of AD by integrating sMRI and fMRI data. Two groups are further separated among MCI subjects, the first group comprises all those subjects who have the probability to progress towards AD and the second group includes all those subjects who have no likelihood to progress towards AD. The classification of MCI subjects as MCI Converter (MCI-C) and MCI Non-Convertor (MCI -NC) was a three-phase procedure. In the feature extraction phase, two types of feature measures were considered, and those include Graph measures based on FC extracted from fMRI and cortical and subcortical measurements obtained from sMRI. In the filter and wrapper feature selection phase, Minimal Redundancy Maximal Relevance (MRMR) and Sequential Features Collection were utilized sequentially. Finally, in the last step, eight SVMs separate the MCI-C subjects from MCI-NC subjects. The approach classifies subjects by analyzing either single-modality or multi-modality data. For the single modality, it yields the accuracies of 93% for fMRI, 89% for sMRI and 97% accuracy for the multi-modal data [21].

To find the progression of EMCI to LMCI and then to AD, another cluster-based strategy was employed on fMRI data that aggregated the prediction of multiple weighted random SVM classifiers for the final prediction. After applying the nine steps of preprocessing, the Posterior Cingulate Cortex based FC was extracted as features. Binary classification of EMCI vs LMCI and LMCI vs AD achieved the accuracy of 90% and 88.98% respectively [51].

Jie B [53] developed a novel Weighted Correlation Kernel (WCK) based CNN architecture that extracts high to low-level Dynamic Connectivity Networks (DCNs) as features. After extracting DCNs using WCK, three layers were defined for the extraction of hierarchical features including local, global, and temporal features. The WCK-CNN model performed multi-class classification of AD, CN, EMCI, and LMCI with an accuracy of 57% without relying on traditional FC-based features. In addition to the multi-class classification, another study utilized CNN architecture to analyze the fMRI data. For the diagnosis of AD, CN, EMCI, LMCI, and Significant Memory Concern (SMC), the author first preprocessed the data, and then converted the data into 2-D images. Furthermore, feature extraction, selection, and classification of 2-D images were performed by using AlexNet pre-trained CNN model that achieved the accuracy of 97.63% for multi-class classification [54].

A study fine-tuned a transfer learning-based CNN variant known as MobileNet to classify five categories: CN, MCI, EMCI, LMCI, and AD, achieving an accuracy of 96.6% based on 1,101 images from 300 subjects [55]. Nevertheless, including MCI along with its sub-stages (EMCI and LMCI) presents conceptual inconsistencies. Another investigation utilized a custom-made CNN architecture for CN, MCI, and AD classifications, yielding 89.21% accuracy [52]. They employed the CNN in 2D and 3D domain. However, employing a 2D CNN for sequential data is logically invalid. A further study analyzed the effects of batch normalization and dropout layers in CNN in early AD classification. The findings indicated that minimal or none value of dropout rate leads to better performance [56]. Consequently, this study establishes a dropout rate of 0.25, which has been shown to yield optimal results.

Previous studies performed classification either based on features extracted from connectivity measures or by applying a voting algorithm for final prediction. According to the voting algorithm, 3D-fMRI data belongs to a category if its 2D scans mostly belong to that category. However, Liu M [57] proposed a CNN and RNN-based framework that first extracted the features of each slice using CNN, and afterwards passed them to a Bidirectional Gated Recurrent Unit (BGRU) for feature extraction between multiple slices followed by ensemble classification. For the input of CNN, 3D-PET data is first converted to 2D-image slices. The CNN-LSTM performed multiple binary classifications, AD vs CN with 91.2% accuracy and MCI vs CN with an accuracy of 78.9%.

The CNN-LSTM-based framework proposed in this paper achieved superior results compared to other recent studies in the field of binary and multi-class classification using fMRI data as discussed earlier. While previous approaches have utilized various techniques such as SVM, Naive Bayes, and cluster-based strategies, our framework outperformed them in terms of accuracy and classification performance.

In the binary classification task, the proposed model demonstrated remarkable accuracy, surpassing the results of the other approaches. Our model achieved accuracy above 99% for binary classification tasks, outperforming previous studies where the accuracies achieved ranged from 92% to 94%. Additionally, in multi-class classification tasks, our framework consistently achieved high accuracies, surpassing the results reported in recent studies. Our model achieved an accuracy of 98.99% in classifying AD, MCI, and CN, outperforming other studies that reported accuracies in the range of 57% to 97.63%.

Moreover, the proposed framework demonstrated robustness and effectiveness in dealing with complex classification tasks involving multiple classes.

Our proposed CNN-LSTM framework presents a distinctive approach that advances beyond existing neuroimaging methodologies. Even though, CNN-LSTM architectures have been explored in other domains, our approach introduces a uniquely configured architecture that has not been applied in any context. Unlike existing methods, our framework employs a dual-layer feature learning mechanism, autonomously extracting intra-volume spatial characteristics and inter-volume temporal dynamics. This integrated feature learning approach provides a comprehensive representation of fMRI data, essential for precise classification. By incorporating both CNN and LSTM layers, our model enhances not only classification performance but also demonstrates substantial improvements in feature extraction and temporal analysis by effectively extracting intra-volume and inter-volume features from fMRI data. Furthermore, the model’s ability to achieve high accuracy and robust performance across various classification tasks highlights its efficacy compared to existing approaches in the field.

The proposed CNN-LSTM model consistently achieved outstanding performance across binary and multi-class classification tasks, attaining AUC values of 99.5% or above for most experiments. These high AUC scores demonstrate the model’s ability to discriminate between different stages of cognitive impairment. Similarly, sensitivity and specificity values remained balanced at 99%, indicating both strong true positive and true negative rates, respectively. Compared to prior studies, which mostly reported only accuracy, our model offers a more robust and clinically interpretable performance profile.

### Limitations and constraints of the proposed methodology

While our CNN-LSTM model demonstrates promising performance in Alzheimer’s Disease classification using fMRI data, certain limitations should be acknowledged for broader applicability in clinical and research contexts.

**1. Scanner Variability:** In developing our model, we selected a subset of the ADNI dataset that incorporates data from multiple scanners to increase robustness against scanner-related variability. However, variations in imaging protocols across different institutions may still affect model generalizability. To enhance its adaptability to broader clinical environments, retraining or fine-tuning on external datasets with distinct scanning parameters may be necessary.

**2. Population Diversity:** Although our selected data from ADNI includes participants from varied demographic backgrounds within North America, broader diversity in terms of ethnicity, cultural context, and geographic representation may still be limited. Future studies could further validate the model on datasets that encompass a more globally diverse population to confirm its generalizability across diverse demographic groups.

**3. Dataset Selection and Potential Biases:** Given the ADNI dataset’s large scale, we used a subset of the data to balance computational feasibility with diversity considerations, selecting data across different scanners and participant demographics where possible. However, the model may still reflect inherent biases in the data due to variations in disease progression and cognitive baselines. To mitigate this, future work could incorporate transfer learning, where the model is fine-tuned on additional datasets with broader population representation and clinical variations.

**4. External Validation and Generalizability:** In this study, the model was developed and evaluated exclusively on the ADNI dataset. Although the ADNI dataset is widely used and includes data from multiple sites and scanners, validating the model on a completely independent dataset would provide a more comprehensive assessment of its generalizability. Without this step, there may be limitations in the model’s ability to achieve similar levels of accuracy and effectiveness on data from other sources, particularly where imaging protocols or participant demographics differ significantly. Future research could incorporate external validation on datasets beyond ADNI to better evaluate the model’s robustness across diverse data environments. In scenarios where obtaining a separate dataset is not feasible, techniques such as domain adaptation or transfer learning could help the model adapt to new datasets with minimal re-training. This approach would enhance its applicability to broader clinical settings while maintaining accuracy. By addressing the need for external validation, we aim to clarify the scope and limitations of our model’s current evaluation and provide directions for future work to improve generalizability across datasets.

## Conclusion

This paper proposes an innovative approach for utilizing fMRI data for the identification of AD and its prodromal stages without segmentation and extraction of functional connectivity measures. The primary contribution of this study is the classification of time series data for each subject. As CNN is unable to handle the sequential data, therefore, an integrated framework of CNN and LSTM model was proposed. The proposed approach performs the classification in three phases. In the first phase, intra-volume features are extracted using 18 layers CNN without softmax layer, and then these multiple intra-volume feature vectors of subjects emerge as a stack. The next phase is the selection of optimal features from multiple feature vectors of a subject; these inter-volume features are extracted using LSTM and classified by the softmax layer, followed by a dropout and dense layer. For the evaluation of this study, a subset of 413 participants has been acquired from ADNI that subsume 66 AD, 140 CN, 34 MCI, 91 EMCI and 80 LMCI participants. The proposed CNN-LSTM model has been validated over multiple 2-class, 3-class, and 4-class classifications utilizing ten-fold stratified cross-validation. Consequently, it achieved the highest accuracy of above 99% for the majority of the classification.

In future, we plan to investigate the model’s applicability to data obtained from various scanners and across diverse population groups. We plan to incorporate PET and sMRI data along with their metadata in future. This will allow us to assess the model’s robustness and generalizability, ensuring that it performs consistently well across different imaging conditions and demographic variations. We also plan to expand this study by conducting extensive experiments with different hyperparameters, including dropout rates and learning rates, to evaluate their impact on the overall effectiveness of our model. We’ll also validate our model with separate dataset, other than ADNI study, to evaluate the generalizability of the model.

## Acknowledgment

The authors are also thankful to AIDA Lab CCIS Prince Sultan University, Riyadh, Saudi Arabia, for their support.

## References

[1] Jie B, Liu M, Shen D. Integration of temporal and spatial properties of dynamic connectivity networks for automatic diagnosis of brain disease. Med Image Anal. 2018;47:81–94. doi: 10.1016/j.media.2018.03.013 29702414 PMC5986611

[2] Burns A, Jacoby R, Luthert P, Levy R. Cause of death in Alzheimer’s disease. Age Ageing. 1990;19(5):341–4. 2251969 10.1093/ageing/19.5.341

[3] Alzheimer’s Association. Alzheimer’s disease facts and figures. Alzheimer’s Dementia. 2024;20(5):3708–821. 10.1002/alz.13809 PMC11095490 38689398

[4] Heron M. Deaths: Leading causes for 2016 ; 2018.

[5] Hebert LE, Weuve J, Scherr PA, Evans DA. Alzheimer disease in the United States 2010 -2050) estimated using the 2010 census. Neurology. 2013;80(19):1778–83. doi: 10.1212/WNL.0b013e31828726f5 23390181 PMC3719424

[6] Petersen RC. Mild cognitive impairment. Continuum (Minneap Minn). 2016;22(2 Dementia):404–18. 27042901 10.1212/CON.0000000000000313 PMC5390929

[7] Cui X, Xiang J, Guo H, Yin G, Zhang H, Lan F, et al. Classification of Alzheimer’s disease, mild cognitive impairment, and normal controls with subnetwork selection and graph kernel principal component analysis based on minimum spanning tree brain functional network. Front Comput Neurosci. 2018;12:31. doi: 10.3389/fncom.2018.00031 29867424 PMC5954113

[8] Lee P, Ryoo H, Park J, Jeong Y, Alzheimer’s Disease Neuroimaging Initiative. Morphological and microstructural changes of the hippocampus in early MCI: A study utilizing the Alzheimer’s disease neuroimaging initiative database. J Clin Neurol. 2017;13(2):144–54. doi: 10.3988/jcn.2017.13.2.144 28176504 PMC5392456

[9] Liu S, Cai W, Liu S, Zhang F, Fulham M, Feng D, et al. Multimodal neuroimaging computing: A review of the applications in neuropsychiatric disorders. Brain Inform. 2015;2(3):167–80. doi: 10.1007/s40708-015-0019-x 27747507 PMC4737664

[10] Busato A, Fumene Feruglio P, Parnigotto PP, Marzola P, Sbarbati A. In vivo imaging techniques: A new era for histochemical analysis. Eur J Histochem. 2016;60(4):2725. doi: 10.4081/ejh.2016.2725 28076937 PMC5159782

[11] Farhan S, Fahiem MA, Tahir F, Tauseef H. A comparative study of neuroimaging and pattern recognition techniques for estimation of Alzheimer’s disease. 2013.

[12] Cauda F, D’Agata F, Sacco K, Duca S, Geminiani G, Vercelli A. Functional connectivity of the insula in the resting brain. Neuroimage. 2011;55(1):8–23. doi: 10.1016/j.neuroimage.2010.11.049 21111053

[13] Mohri M, Rostamizadeh A, Talwalkar A. Foundations of machine learning; 2018.

[14] Camacho DM, Collins KM, Powers RK, Costello JC, Collins JJ. Next-generation machine learning for biological networks. Cell. 2018;173(7):1581–92. doi: 10.1016/j.cell.2018.05.015 29887378

[15] Chaves R, Ramírez J, Górriz JM, López M, Salas-Gonzalez D, Alvarez I, et al. SVM-based computer-aided diagnosis of the Alzheimer’s disease using t-test NMSE feature selection with feature correlation weighting. Neurosci Lett. 2009;461(3):293–7. doi: 10.1016/j.neulet.2009.06.052 19549559

[16] Beheshti I, Demirel H, Matsuda H, Alzheimer’s Disease Neuroimaging Initiative. Classification of Alzheimer’s disease and prediction of mild cognitive impairment-to-Alzheimer’s conversion from structural magnetic resource imaging using feature ranking and a genetic algorithm. Comput Biol Med. 2017;83:109–19. doi: 10.1016/j.compbiomed.2017.02.011 28260614

[17] Cabral C, Silveira M, Alzheimer’s Disease Neuroimaging Initiative. Classification of Alzheimer’s disease from FDG-PET images using favourite class ensembles. Annu Int Conf IEEE Eng Med Biol Soc. 2013;2013:2477–80. doi: 10.1109/EMBC.2013.6610042 24110229

[18] Tong T, Gray K, Gao Q, Chen L, Rueckert D. Multi-modal classification of Alzheimer’s disease using nonlinear graph fusion. Pattern Recogn. 2017;63:171–81. doi: 10.1016/j.patcog.2016.10.009

[19] Samper-González J, Burgos N, Bottani S, Fontanella S, Lu P, Marcoux A, et al. Reproducible evaluation of classification methods in Alzheimer’s disease: Framework and application to MRI and PET data. Neuroimage. 2018;183:504–21. doi: 10.1016/j.neuroimage.2018.08.042 30130647

[20] Samper-González J. Yet another ADNI machine learning paper? Paving the way towards fully-reproducible research on classification of Alzheimer’s disease. Springer; 2017. p. 53–60.

[21] Hojjati SH, Ebrahimzadeh A, Khazaee A, Babajani-Feremi A, Alzheimer’s Disease Neuroimaging Initiative. Predicting conversion from MCI to AD by integrating rs-fMRI and structural MRI. Comput Biol Med. 2018;102:30–9. doi: 10.1016/j.compbiomed.2018.09.004 30245275

[22] Khazaee A, Ebrahimzadeh A, Babajani-Feremi A. Application of advanced machine learning methods on resting-state fMRI network for identification of mild cognitive impairment and Alzheimer’s disease. Brain Imaging Behav. 2016;10(3):799–817. doi: 10.1007/s11682-015-9448-7 26363784

[23] Litjens G, Kooi T, Bejnordi BE, Setio AAA, Ciompi F, Ghafoorian M, et al. A survey on deep learning in medical image analysis. Med Image Anal. 2017;42:60–88. doi: 10.1016/j.media.2017.07.005 28778026

[24] Islam J, Zhang Y. A novel deep learning based multi-class classification method for Alzheimer’s disease detection using brain MRI data. Springer. 2017. p. 213–22.

[25] Korolev S, Safiullin A, Belyaev M, Dodonova Y. Residual and plain convolutional neural networks for 3D brain MRI classification. In: 2017 IEEE 14th international symposium on biomedical imaging (ISBI 2017), 2017. 835–8. 10.1109/isbi.2017.7950647

[26] Hosseini-Asl E, Ghazal M, Mahmoud A, Aslantas A, Shalaby AM, Casanova MF, et al. Alzheimer’s disease diagnostics by a 3D deeply supervised adaptable convolutional network. Front Biosci (Landmark Ed). 2018;23(3):584–96. doi: 10.2741/4606 28930562

[27] Qureshi MNI, Ryu S, Song J, Lee KH, Lee B. Evaluation of functional decline in Alzheimer’s dementia using 3D Deep learning and group ICA for rs-fMRI measurements. Front Aging Neurosci. 2019;11:8. doi: 10.3389/fnagi.2019.00008 30804774 PMC6378312

[28] Hon M, Khan N. Towards Alzheimer’s disease classification through transfer learning; 2017.

[29] Chiang C-H, Weng C-L, Chiu H-W. Automatic classification of medical image modality and anatomical location using convolutional neural network. PLoS One. 2021;16(6):e0253205. doi: 10.1371/journal.pone.0253205 34115822 PMC8195382

[30] Kim M-J, Kim J-H. Development of convolutional neural network model for classification of cardiomegaly X-ray images. J Mech Med Biol. 2022;22(08). doi: 10.1142/s0219519422400206

[31] Maruyama T, Hayashi N, Sato Y, Hyuga S, Wakayama Y, Watanabe H, et al. Comparison of medical image classification accuracy among three machine learning methods. J Xray Sci Technol. 2018;26(6):885–93. doi: 10.3233/XST-18386 30223423

[32] Lakhani P. The importance of image resolution in building deep learning models for medical imaging. Radiol Artif Intell. 2020;2(1):e190177. doi: 10.1148/ryai.2019190177 33939779 PMC8017377

[33] Gu J, Wang Z, Kuen J, Ma L, Shahroudy A, Shuai B, et al. Recent advances in convolutional neural networks. Pattern Recogn. 2018;77:354–77. doi: 10.1016/j.patcog.2017.10.013

[34] Hagan M, Demuth H, Beale M, Jesús OD. Neural network design; 1996.

[35] Haykin S. Neural networks; 1994.

[36] Bengio Y, Simard P, Frasconi P. Learning long-term dependencies with gradient descent is difficult. IEEE Trans Neural Netw. 1994;5(2):157–66. doi: 10.1109/72.279181 18267787

[37] Hochreiter S. The vanishing gradient problem during learning recurrent neural nets and problem solutions. Int J Unc Fuzz Knowl Based Syst. 1998;06(02):107–16. doi: 10.1142/s0218488598000094

[38] Hochreiter S, Schmidhuber J. Long short-term memory. Neural Computat. 1997;9(8):1735–80. 10.1162/neco.1997.9.8.1735 9377276

[39] Stratified cross validation. Encyclopedia of Machine Learning and Data Mining. Boston, MA: Springer US; 2017. p. 1191.

[40] Wu X, Li J, Ayutyanont N, Protas H, Jagust W, Fleisher A, et al. The receiver operational characteristic for binary classification with multiple indices and its application to the neuroimaging study of Alzheimer’s disease. IEEE/ACM Trans Comput Biol Bioinform. 2013;10(1):173–80. doi: 10.1109/TCBB.2012.141 23702553 PMC4085147

[41] Unknown. ROC Curve. Encyclopedia of machine learning and data mining. Boston, MA: Springer US; 2017. p. 1116.

[42] Zeugmann T. Boston, MA: Springer US; 2011. p. 781.

[43] Kingma DP, Ba J. Adam: A method for stochastic optimization; 2014.

[44] Bi X, Jiang Q, Sun Q, Shu Q, Liu Y. Analysis of Alzheimer’s disease based on the random neural network cluster in fMRI. Front Neuroinform. 2018;12:60. 30245623 10.3389/fninf.2018.00060 PMC6137384

[45] Bi X-A, Shu Q, Sun Q, Xu Q. Random support vector machine cluster analysis of resting-state fMRI in Alzheimer’s disease. PLoS One. 2018;13(3):e0194479. doi: 10.1371/journal.pone.0194479 29570705 PMC5865739

[46] Khazaee A, Ebrahimzadeh A, Babajani-Feremi A. Identifying patients with Alzheimer’s disease using resting-state fMRI and graph theory. Clin Neurophysiol. 2015;126(11):2132–41. doi: 10.1016/j.clinph.2015.02.060 25907414

[47] Khazaee A, Ebrahimzadeh A, Babajani-Feremi A, Alzheimer’s Disease Neuroimaging Initiative. Classification of patients with MCI and AD from healthy controls using directed graph measures of resting-state fMRI. Behav Brain Res. 2017;322(Pt B):339–50. doi: 10.1016/j.bbr.2016.06.043 27345822

[48] Nguyen DT, Ryu S, Qureshi MNI, Choi M, Lee KH, Lee B. Hybrid multivariate pattern analysis combined with extreme learning machine for Alzheimer’s dementia diagnosis using multi-measure rs-fMRI spatial patterns. PLoS One. 2019;14(2):e0212582. doi: 10.1371/journal.pone.0212582 30794629 PMC6386400

[49] Sarraf S, DeSouza DD, Anderson J, for the Alzheimer’s Disease Neuroimaging Initiative GT. DeepAD: Alzheimer’s disease classification via deep convolutional neural networks using MRI and fMRI. bioRxiv; 2017. p. 070441.

[50] Pei S, Guan J, Zhou S. Classifying early and late mild cognitive impairment stages of Alzheimer’s disease by fusing default mode networks extracted with multiple seeds. BMC Bioinform. 2018;19(Suppl 19):523. doi: 10.1186/s12859-018-2528-0 30598074 PMC6311889

[51] Bi X, Xu Q, Luo X, Sun Q, Wang Z. Analysis of progression toward Alzheimer’s disease based on evolutionary weighted random support vector machine cluster. Front Neurosci. 2018;12:716. 30349454 10.3389/fnins.2018.00716 PMC6186825

[52] Tufail AB, Anwar N, Othman MTB, Ullah I, Khan RA, Ma Y-K, et al. Early-stage Alzheimer’s disease categorization using PET neuroimaging modality and convolutional neural networks in the 2D and 3D domains. Sensors (Basel). 2022;22(12):4609. doi: 10.3390/s22124609 35746389 PMC9230850

[53] Jie B, Liu M, Lian C, Shi F, Shen D. Developing novel weighted correlation kernels for convolutional neural networks to extract hierarchical functional connectivities from fMRI for disease diagnosis. In: Machine learning in medical imaging: 9th international workshop, MLMI 2018, held in conjunction with MICCAI 2018, Granada, Spain, September 16, 2018, Proceedings 9, 2018. 1–9. 10.1007/978-3-030-00919-9_1 PMC6410567 30868142

[54] Kazemi Y, Houghten S. A deep learning pipeline to classify different stages of Alzheimer’s disease from fMRI data. In: 2018 IEEE conference on computational intelligence in bioinformatics and computational biology (CIBCB); 2018. p. 1–8. 10.1109/cibcb.2018.8404980

[55] Mohi ud din dar G, Bhagat A, Ansarullah SI, Othman MTB, Hamid Y, Alkahtani HK, et al. A novel framework for classification of different Alzheimer’s disease stages using CNN model. Electronics. 2023;12(2):469. doi: 10.3390/electronics12020469

[56] Tufail AB, Ullah I, Rehman AU, Khan RA, Khan MA, Ma Y-K, et al. On disharmony in batch normalization and dropout methods for early categorization of Alzheimer’s disease. Sustainability. 2022;14(22):14695. doi: 10.3390/su142214695

[57] Liu M, Cheng D, Yan W, Alzheimer’s Disease Neuroimaging Initiative. Classification of Alzheimer’s disease by combination of convolutional and recurrent neural networks using FDG-PET images. Front Neuroinform. 2018;12:35. doi: 10.3389/fninf.2018.00035 29970996 PMC6018166

